# Anti-apoptotic effect of dexamethasone in an ototoxicity model

**DOI:** 10.1186/s40824-017-0090-x

**Published:** 2017-04-06

**Authors:** Jin Ho Lee, Se Heang Oh, Tae Ho Kim, Yoon Young Go, Jae-Jun Song

**Affiliations:** 1 Department of Advanced Materials, Hannam University, Daejeon, Korea; 2 Department of Nanobiomedical Science & WCU Research Center, Dankook University, Cheonan, Korea; 3 Department of Otorhinolaryngology-Head and Neck Surgery, Korea University College of Medicine, Seoul, Korea

**Keywords:** Explant culture, Gentamicin, Ototoxicity, Dexamethasone

## Abstract

**Background:**

Dexamethasone (DEX) is used for the treatment of various inner ear diseases. However, the molecular mechanism of DEX on gentamicin induced hair cell damage is not known. Therefore, this study investigated the protective effect of DEX on gentamicin (GM)-induced ototoxicity and the effect of GM on the expression of apoptosis related genes.

**Methods:**

The protective effects of DEX were measured by phalloidin staining of explant cultures of organ of Corti from postnatal day 2–3 mice with GM-induced hair cell loss. Terminal deoxynucleotidyl transferase dUTP nick end labeling staining was used to detect apoptosis and immunofluorescence was done to analyze the effect of DEX on the expression of apoptosis related genes.

**Results:**

Cochlear explant cultures of postnatal day-4-old mice were exposed to 0, 1, 5, 10, 30, 50, and 100 μg/ml DEX and GM during culture. DEX protected from GM-induced hair cell loss in the inner ear of postnatal day 4 mice. To understand the molecular mechanisms by which DEX pre-treatment decreased hair cell loss, the testes of cochlear explant cultures of postnatal day 4 mice were examined for changes in expression of cochlear apoptosis mediators. The pro-apoptotic protein Bax was significantly down-regulated and numbers of apoptotic hair cells were decreased.

**Conclusions:**

DEX has a protective effect on GM-induced hair cell loss in neonatal cochlea cultures and the protective mechanism may involve inhibition of the mitochondrial apoptosis pathway. The combination with scaffold technique can improve delivery of DEX into the inner ear to protect GM-induced ototoxicity.

## Background

Aminoglycosides remain important antibiotics in current clinical practice and are widely used globally. Their antibacterial effect includes enterococcus, mycobacteria, and especially multi-drug-resistant Gram-negative bacteria [1]. However, their clinical usefulness is limited by their ototoxicity and nephrotoxicity [2]. Aminoglycosides cause ototoxicity in 2–25% of patients and if high-frequency hearing loss is tested, half of all patients may be affected [3]. The exact mechanism of GM-induced ototoxicity is unclear. The main signal transduction pathway of hair cell damage is ROS production and resulting apoptosis [1, 4]. One signaling pathway activated by aminoglycosides via ROS is the c-Jun N-terminal kinase (JNK) pathway, which contributes to cell apoptosis [5]. One of the downstream targets of JNK is the transcription factor, activating protein-1, the main component of which is c-Fos protein [6].

Gentamicin (GM), which is one of the most commonly used aminoglycoside antibiotics, can induce hearing loss and balance disturbance due to the destruction of cochlear and vestibular hair cells [7]. The exact mechanism of GM-induced ototoxicity is unclear. The main signal transduction pathway of hair cell damage involves reactive oxygen species (ROS) production and resulting apoptosis [1, 4]. Protection of hair cell damage is an important target for the prevention of sensory neural hearing loss. However, few clinical agents are available for the treatment of ototoxicity-related sensory neural hearing loss.

Dexamethasone (DEX) is a synthetic steroid analog used for the treatment of various inner ear diseases including sudden idiopathic sensory neural hearing loss, Meniere’s disease and Bell’s palsy. However, the exact biological mechanism of DEX is unclear. In animal models, intratympanic administration of DEX protected against cisplatin-induced ototoxicity [8, 9]. However, the molecular mechanism of protective effect of DEX on GM-induced hair cell damage is not known. DEX improves oxidative energy metabolism of brain mitochondria and stimulates ATP synthesis by increasing mitochondrial enzymes [10, 11]. Mitochondria are the primary site of oxidative metabolism and apoptosis. The intrinsic pathway of apoptosis is activated by cytochrome c release from mitochondria, mediated by the opening of the mitochondrial permeability pore [12].

We hypothesized that DEX can lessen apoptosis by modulating the activity of apoptosis-related genes, which in turn modulates the mitochondrial membrane permeability to molecules including Bax. The purpose of this study was to investigate the protective effect of DEX on GM-induced ototoxicity and the effect of DEX on the expression of apoptosis-related genes.

## Methods

### Explant model

Cochlea organotypic cultures were made as described previously [13]. All protocols were in accordance with the guidelines of the Animal Care Ethics Committee of Dongguk University Ilsan Hospital guidelines. ICR mouse (Koatech, Pyeongtaek, Gyeonggi, Korea) pups were decapitated on postnatal day 4. The cochlea of each mouse was carefully dissected as a flat-surface preparation of the organ of Corti. A drop of 3.86 mg/mL Type I rat tail collagen (BD Biosciences, Piscataway, NJ, USA) was added to a solution containing 0.02 N acetic acid, 10x basal medium Eagle’s medium (Sigma-Aldrich, St. Louis, MO, USA), and 2% sodium carbonate in a 9:1:1 ratio in a 35 mm-diameter culture dish (Nunc, Rochester, NY, USA) and allowed to gel. Afterwards, 1 mL of serum-free Dulbecco’s modified Eagle’s medium (Welgene, Daegu, Korea) supplemented with 1% N-1 supplement (Sigma-Aldrich) and 50 U/mL penicillin G (Sigma-Aldrich) was added. The organ of Corti was gently pressed onto the surface of the collagen gel with forceps and held in place by the surface tension of the culture medium. Cochlea cultures were incubated at 37°C in a humidified atmosphere of 5% CO_2_ overnight. Various concentrations of DEX were used in a 24-h pre-treatment prior to a 24-h exposure to a medium containing 0.3 mM GM.

After incubation for 24 h, the cultures were fixed, permeabilized, and stained with Alexa Fluor 350 phalloidin (Invitrogen, Carlsbad, CA, USA). The explants were then mounted on a slide with Flurogel in tris buffer (Electron Microscopy Sciences, Hatfield, PA, USA), coverslipped, and examined with a DP70 fluorescence microscope (Olympus, Tokyo, Japan). Each labeled hair cell cilia was counted in the three outer hair cells (OHC) rows and one inner hair cell (IHC) row. Cells were considered to be missing if there was a gap in the normal geometric array and no stereocilia or cuticular plates were apparent. The number of cells was counted over a distance of 100 μm from five randomly selected fields for the basal, middle, and apical turns of each explant. The average from five fields was considered as a single sample, and typically 5–11 specimens were evaluated for each condition.

### Terminal deoxynucleotidyltransferase dUTP nick end labeling (TUNEL)

The anti-apoptosis effect was assessed using the TUNEL assay. DNA fragmentation was assessed in situ using a fluorescein-based In situ cell death detection kit (Roche, Penzberg, Bayern, Germany) as described by the manufacturer, with minor modifications. Fixed cochlea organotypic cultures were placed in 0.01 M phosphate buffered saline (PBS) for 10 min, and treated with 0.03% Triton X-100 for at least 15 min at room temperature. After washing twice with deionized water for 2 min, each culture was immersed in 1× TdT labeling buffer for 5 min. The cultures were incubated in TUNEL mix containing 50× TdT dNTP, 50× cation (Mg^2+^), 50× TdT enzyme, and 1× TdT labeling buffer for 60 min at 37°C in a humidity chamber. The reaction was terminated by washing in 1× TdT stop buffer for 5 min. The cultures were washed in 0.05% PBS-Tween 20 (PBS-T), before being mounted on glass with coverslips using Tris buffer containing flourogel (Electron Microscopy Sciences). Samples were viewed using a fluorescence microscope using a 495 nm filter.

### Immunofluorescence staining for Bax

For Bax labeling, the cultures were fixed and permeabilized. Nonspecific staining was blocked with 2.5% normal donkey serum (Sigma-Aldrich) for 30 min at room temperature. The cultures were then incubated with Bax rabbit polyclonal IgG (200 μg/mL, 1:200 dilution; Santa Cruz Biotechnology, Santa Cruz, CA, USA) in PBS for 24 h at 4°C followed by Alexa Fluor® 594 donkey anti-rabbit IgG (1:500 dilution; Molecular Probes, Eugene, OR, USA). All cultures were counterstained with Hoechst 33342 (Molecular Probes) for 3 min. The samples were mounted on a slide with fluorogel (Electron Microscopy Sciences), coverslipped, and examined with a model DP70 fluorescence microscope (Olympus). Samples were viewed using a fluorescence microscope using a 488nm filter and 594 nm filter.

## Results

### Protective effect of DEX on GM-induced hair cell loss

**Fig. 1:**
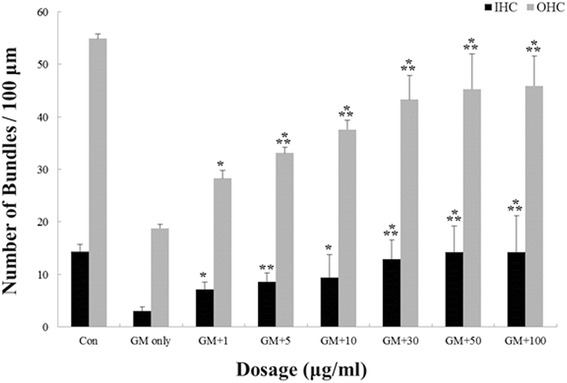
Protective effect of DEX against GM-induced hair cell loss in organotypic cochlear cultures from postnatal mice. GM induced hair cell loss in the control (Con) group and DEX had a significant effect at concentrations higher than 5 μg/mL. *p* < 0.005 as compared with the GM-alone group; *p* < 0.05*, *p* < 0.005**, *p* < 0.0005***)

**Fig. 2:**
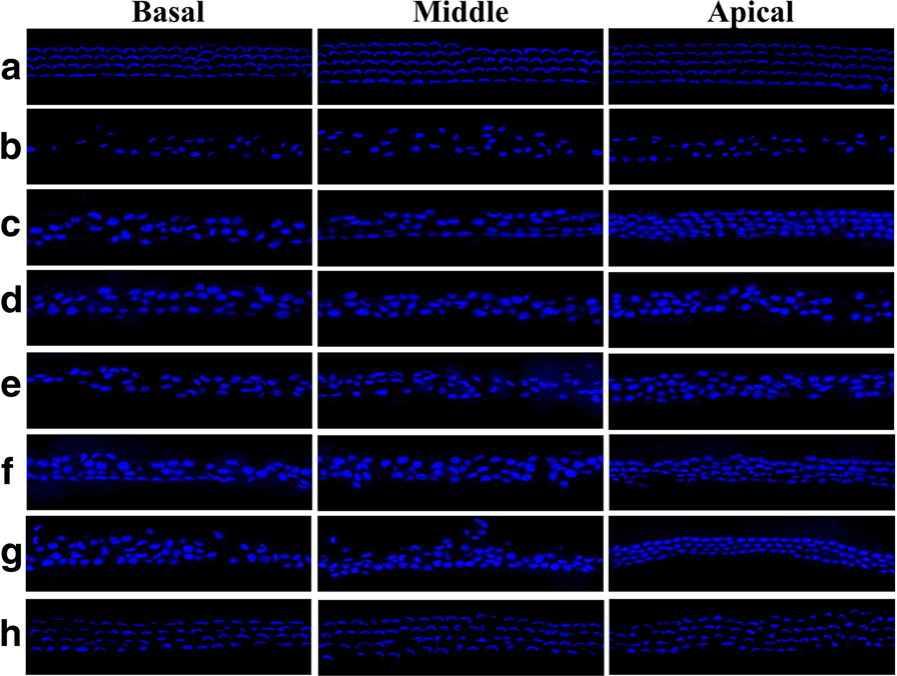
Cochlea histology in mice exposed to 0.3 mM GM and dose-dependence of DEX treatment. Phalloidin stained cochlear explant cultures of postnatal day-4-old mice pretreated with PBS (**a**), 0.3 mM GM only (**b**), 1 μg/mL DEX (**c**), 5 μg/mL DEX (**d**), 10 μg/mL DEX (**e**), 30 μg/mL DEX (**f**), 50 μg/mL DEX (**g**), and 300 μg/mL DEX (**h**) (400x magnification)

Cochlear cultures from postnatal mice were pretreated with medium containing DEX at concentrations of 0, 1, 5, 10, 30, 50, and 100 μg/mL and then exposed to a medium containing 0.3 mM GM for 24 h. The specific concentration of GM (0.3 mM) to affect inner or outer HC loss was determined from our previous works [1, 14]. Compared with the control group, IHCs and OHCs of the organ of Corti in the GM-only group were destroyed. Viable phalloidin-labeled hair cells were counted and shown in Fig. 1. A dose-dependent protective effect of DEX was evident. Compared to use of GM alone, pretreatment with DEX concentrations exceeding 5 ug/mL were significantly protective (*p* < 0.005; Fig. 1). The images of phalloidin stained cochlear explant also showed that DEX pretreatment reduced the GM-induced ototoxicity in a dose-dependent manner. GM alone group severely distorted the anatomy of the organ of corti, compared with untreated controls. However, above 5 ug/mL of DEX effectively provided protection against GM-induced ototoxicity. The alignment of bundles in the organ of Corti was retained their shape by the highest DEX (300 μg/mL) pretreatment in GM-induced ototoxicity (Fig. 2). Taken together, these results indicated that DEX was significantly protective against GM-induced hair cell loss.

### TUNEL assay and Immunofluorescence (IFC)

**Fig. 3:**
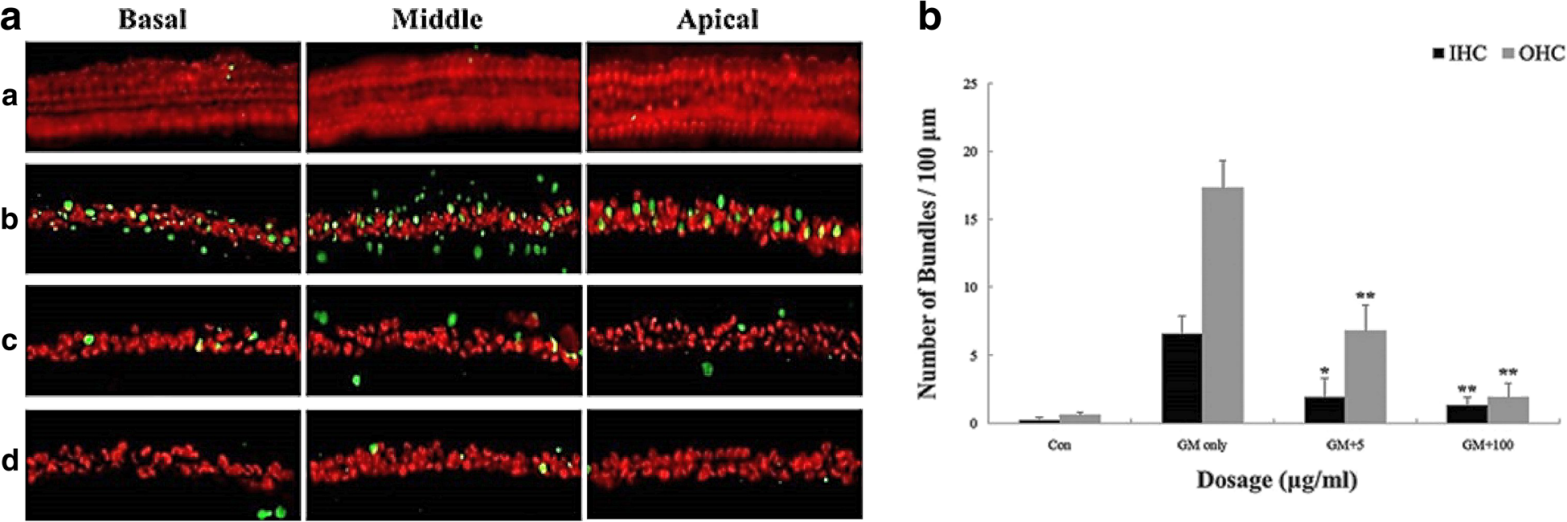
Decreased apoptosis in organotypic cochlear cultures of P4 mice exposed to GM and DEX. A. TUNEL assay was conducted on explant cultures of P4 mice cochlea prepared from groups treated with PBS only (**a**), 0.3 mM GM only (**b**), and following pretreatment with DEX concentrations of 5 μg/mL (**c**), 100 μg/mL (**d**) prior to GM exposure. B. Apoptotic hair cells (green fluorescence) were counted from the cochlear explant cultures of P4 mice. Data are mean ± SEM from three independent tests in each dose of pre-treated DEX with 0.3 mM GM. *p* < 0.05 as compared with the GM-alone group. (*p* < 0.05* and *p* < 0.005**, 400x magnification.)

**Fig. 4:**
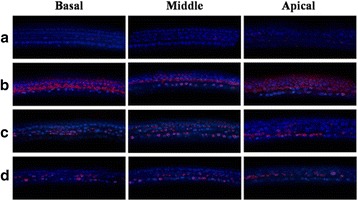
Down-regulated Bax expression (red fluorescence following counterstaining with 4',6-diamidino-2-phenylindole) in P4 mice cochlea treated with low dose and high dose of DEX. Cochlear explant cultures of P4 mice were used for IHC examination for Bax; PBS only (**a**), 0.3 mM GM only (**b**), 5 μg/mL (**c**), and 100 μg/mL (**d**) DEX with 0.3 mM GM (400x magnification)

The influence of DEX pretreatment on cochlear apoptosis induced by GM was assessed using the TUNEL assay. TUNEL-positive cells (green fluorescence following counterstaining with Myosin 7a) were clearly decreased at higher doses of DEX, especially for hair cells (Fig. 3A). DEX pretreatment inhibited GM-induced apoptosis in a dose-dependent manner in IHCs and OHCs in cochlear explants cultures. At concentrations above 5 μg/mL, DEX pretreatment showed an anti-apoptotic effect in IHCs and OHCs (Fig. 3B; p < 0.005). Since pro-apoptotic proteins in the Bcl-2 family are critical in regulating cell death and survival of diverse species, we next assessed if expression of any particular members of the pro-apoptotic Bcl-2 family were affected by DEX. Immunofluorescence analyses for Bax in cochlear explant cultures from P4 mice revealed decreased expression in cochlear hair cells in a DEX dose-dependent manner (Fig. 4).

## Discussion

This study investigated the protective effects of DEX on GM-induced hair cell loss and the expression of apoptosis-related genes in neonatal mouse cochlea cultures. DEX protected against GM-induced hair cells loss in a dose-dependent manner and modulated the expression of Bax.

Many agents include sodium thiosulphate [15], N-acetyl cysteine [16], alpha tocopherol [17], L-methionine [18], and gingko biloba [19] have been studied to find otoprotectants. Glucocorticoids including prednisone, dexamethasone, and methylprednisolone are often used for the treatment of various inner ear diseases including autoimmune inner ear, ehdolymphatic hydrops, Meniere’s disease, tinnitus, and sudden sensorineural hearing loss [20]. Also, DEX protects against various types of inner ear damage including acoustic trauma and ototoxicity [21, 22].

Though the exact molecular mechanism of protective effect of DEX is not known, DEX is thought to function via its glucocorticoid receptor and signal transduction pathway mediating inflammation and apoptosis by the modulation of nuclear factor kappa B (NFkB) in the inner ear [23] and by limiting the formation of ROS in the inner ear [24]. The presence of corticosteroid receptors in the mouse inner ear provides further evidence that steroids can exert an effect on the inner ear [25]. Systemic injection of DEX is reportedly effective in preventing cisplatin-induced ototoxicity [26]. Intratympanic administration of DEX protects against cisplatin-induced ototoxicity in animal models [8, 9, 27, 28]. We have also shown that DEX significantly protect against GM-induced hair cells loss in vivo in a dose-dependent manner (Figs.1 and 2). Especially, the high concentration of DEX (300 μg/mL) showed complete protection against GM-induced ototoxicity (Fig. 2).

In the naïve organ of Corti explants treated with DEX, TNFR1 expression was reduced, Bcl-2 and Bcl-xl expression were increased, and Bax/Bcl-2 ratio was decreased [29]. In our study, the critical concentration of GM affected apoptosis of hair cells in the organ of Corti which was determined using TUNEL assay (Fig. 3). DEX pretreatment significantly reduced GM induced cochlear apoptosis above 5 μg/mL (Fig. 3). In addition, we have evaluated that DEX decreased the expression of apoptotic activator such as Bax when GM induced apoptosis in cochlear hair cells (Fig. 4). GM-induced ototoxicity results in the activation of intrinsic apoptotic pathway mediated by the change of mitochondrial membrane permeability [30]. Bcl2 family protein, a mitochondrial membrane protein, functions as a checkpoint for cell death and survival signals in the mitochondria. These proteins control the permeability of the mitochondrial membrane and leakage of cytochrome-c, which activates the upstream caspase [30]. The balance of pro- and anti-apoptotic proteins of the Bcl2 protein family is important for the initiation of apoptosis. Anti-apoptotic Bcl-2 proteins are able to bind to pro-apoptotic Bcl-2 proteins, neutralizing the pro-apoptotic signal [31]. However, when the balance moves in favor of apoptosis, the pro-apoptotic cytoplasmic Bcl-2 member Bax translocates to the mitochondria, causing pores in the mitochondrial membrane and release of cytchome c. Therefore, our study demonstrated that DEX suppressed apoptosis of hair cells in the cochlear by regulation of Bax expression in GM-induced ototoxicity model.

In the ototoxicity model, *Dinh* et al. has shown that DEX has preventive effect against TNF-α induced ototoxicity in organ of Corti explants [32], suggesting the anti-inflammatory effect of DEX. Future studies need to determine whether DEX can protect against pro-inflammatory pathway associated with GM ototoxicity. In addition, the development of DEX delivery system into the inner ear are also important to investigate as well. For example, combination with scaffold system like DEX-eluting nanoparticles with hydrogel that may be helpful to deliver prolonged levels of DEX in the cochlear as a drug delivery system (DDS).

## Conclusion

We investigated the protective effects of DEX on GM-induced hair cell loss in neonatal mouse cochlea cultures. The pretreatment of DEX decreased the expression of pro-apoptotic Bax protein which might protect the mitochondrial apoptosis pathway on GM-induced ototoxicity. Taken together our study provides the protective mechanism of DEX in an ototoxicity model.

## Abbreviations

DEX: Dexamethasone
GM: Gentamicin
IFC: Immunofluorescence
IHC: Inner hair cells
JNK: c-JUN N-terminal kinase
NFkB: Nuclear factor kappa B
OHC: Outer hair cells
ROS: Reactive oxygen species
TUNEL: Terminal deoxynucleotidyltransferase dUTP nick end labeling
